# No evidence for high-pressure melting of Earth’s crust in the Archean

**DOI:** 10.1038/s41467-019-13547-x

**Published:** 2019-12-05

**Authors:** Robert H. Smithies, Yongjun Lu, Tim E. Johnson, Christopher L. Kirkland, Kevin F. Cassidy, David C. Champion, David R. Mole, Ivan Zibra, Klaus Gessner, Jyotindra Sapkota, Matthew C. De Paoli, Marc Poujol

**Affiliations:** 1 Geological Survey of Western Australia, Department of Mines, Industry Regulation and Safety, 100 Plain Street, East Perth, WA 6004 Australia; 2 School of Earth and Planetary Sciences, The Institute for Geoscience Research (TIGeR), Centre for Exploration Targeting - Curtin Node, Space Science Technology Centre, Curtin University, GPO Box U1987, Perth, WA 6845 Australia; 3 Centre for Global Tectonics, State Key Laboratory of Geological Processes and Mineral Resources, China University of Geosciences, Wuhan, Hubei Province 430074 China; 4 Bare Rock Geological Services Pty Ltd, PO Box 1633, Fremantle, WA 6959 Australia; 5 Geoscience Australia, GPO Box 378, Canberra, ACT 2601 Australia; 6 Centre for Exploration Targeting and Australian Research Council Centre of Excellence for Core to Crust Fluid Systems (CCFS), School of Earth Sciences, The University of Western Australia, Crawley, WA 6009 Australia; 7 Mineral Exploration Research Centre (MERC), Harquail School of Earth Sciences and Goodman School of Mines, Laurentian University, Sudbury, Ontario, P3E 2C6 Canada; 8 Univ Rennes, CNRS, Géosciences Rennes - UMR 6118, 35000 Rennes, France

**Keywords:** Geochemistry, Geodynamics, Tectonics

## Abstract

Much of the present-day volume of Earth’s continental crust had formed by the end of the Archean Eon, 2.5 billion years ago, through the conversion of basaltic (mafic) crust into sodic granite of tonalite, trondhjemite and granodiorite (TTG) composition. Distinctive chemical signatures in a small proportion of these rocks, the so-called high-pressure TTG, are interpreted to indicate partial melting of hydrated crust at pressures above 1.5 GPa (>50 km depth), pressures typically not reached in post-Archean continental crust. These interpretations significantly influence views on early crustal evolution and the onset of plate tectonics. Here we show that high-pressure TTG did not form through melting of crust, but through fractionation of melts derived from metasomatically enriched lithospheric mantle. Although the remaining, and dominant, group of Archean TTG did form through melting of hydrated mafic crust, there is no evidence that this occurred at depths significantly greater than the ~40 km average thickness of modern continental crust.

## Abstract (web summary)

Some of Earth’s earliest continental crust has been previously inferred to have formed from partial melting of hydrated mafic crust at pressures above 1.5 GPa (more than 50 km deep), pressures typically not reached in post-Archean continental crust. Here, the authors show that such high pressure signatures can result from melting of mantle sources rather than melting of crust, and they suggest there is a lack of evidence that Earth’s earliest crust melted at depths significantly below 40 km.

## Introduction

**Fig. 1:**
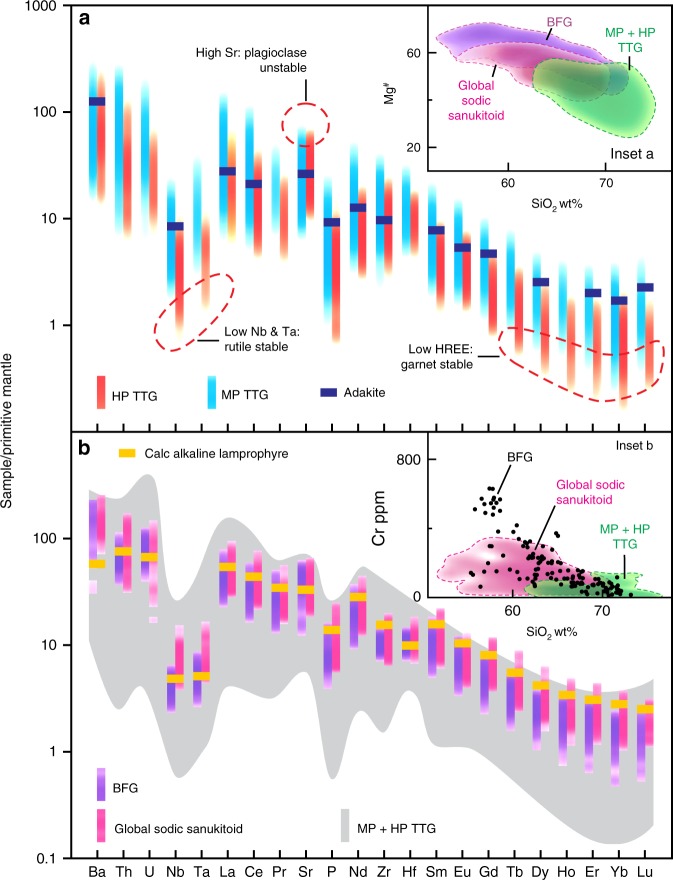
Trace element patterns of the Black Flag Group (BFG) compared to those of other Archean sodic magmas. Mantle-normalized^57^ trace element diagrams comparing; **a** high pressure (HP) and medium pressure (MP) rocks of the tonalite–trondhjemite–granodiorite (TTG)^9^ composition; **b** rocks of the Black Flag Group and global sodic sanukitoids^58^. The data are shown as kernel density strips after removing 10% outliers (see Methods). Also shown is the average composition of high-Si adakite^26^ and of calc-alkaline lamprophyre dykes in the stratigraphy underlying the BFG. Insets a and b: variation in Mg^#^ and Cr (inset a and b, respectively) with SiO_2_ (calculated volatile free) for TTG (combined MP and HP), global sodic sanukitoids and rocks of the BFG. Dashed lines outline kernel density plots comprising 90% of the data. Sanukitoid series ideally should be dominated by samples having Mg^#^ >60 and Cr >200 ppm at 60 wt% SiO_2_ (ref. ^25^). Whilst some of the global sodic sanukitoids satisfy this criterion, most of BFG rocks have even greater Mg^#^ and Cr.

Silica-rich (felsic) granitic rocks, the dominant rock type of Earth’s continental crust, exhibit a wide range of chemical compositions that are indicative of their origin. Within the range of crustal melting that produces voluminous granitic rocks, variations in K_2_O/Na_2_O ratios mainly reflect variations in source composition. The dominance of sodic (i.e., K_2_O/Na_2_O < 0.6) granites of the tonalite–trondhjemite–granodiorite (TTG) series, particularly in the Archean, reflects the fact that most of these were derived from a hydrated basaltic source^1,2^, and this transformation to felsic compositions^3–6^ accounts for as much as 70%^(5)^ of the Earth’s current volume of continental crust. Melting of these source rocks occurred under a range of pressures (depths) that can be monitored through changes in trace element ratios that are controlled by various pressure-sensitive minerals. Most important are Sr/Y and La/Yb ratios, which rapidly increase at pressures above which melting leaves increasingly abundant garnet (sequesters Y and Yb and other heavy rare earth elements (HREE)) but decreasing amounts of plagioclase (sequesters Sr) in the residual source. Thus, it is recognized that melting mafic crust more enriched in incompatible trace elements such as La, Th, Sr and K^1,7,8^, but otherwise compositionally similar to average modern mid-oceanic ridge basalt, at pressures >1.0 GPa, produces sodic felsic melts enriched in Sr and La but depleted in Y and Yb, with Sr/Y and La/Yb both >40, similar to many TTG^1,8–10^ (Fig. 1a). However, significant volumes of compositionally appropriate Archean source material have not been identified^7,11^.

The difficulty in separating the competing effects of source enrichment from melting pressures are well known^6,12^. However, with broad assumptions on the composition of the mafic source, experimental data and trace element modeling permit a distinction between medium-pressure (MP) TTG, complying with compositional ranges expected for melts leaving plagioclase-poor, garnet-rich, amphibolite residues at 1.0–1.5 GPa (depths of 35–50 km; >60% of global TTG^9^), and less common high-pressure (HP) TTG, leaving rutile-bearing eclogite residues at >2.0 GPa (>70 km; <25% of global TTG)^9^ (Fig. 1a). Recent studies have demonstrated the sensitivity of garnet stability to the Mg^#^ (100 × atomic Mg/(Mg + Fe) of the source rock, and have shown that MP TTG may be stable to pressures as low as 0.7 GPa^13^ (~25) km, well within the depths of modern continental crust (35–40 km^14,15^). However, conditions inferred for formation of HP TTG require crustal melting at extreme depths, seen today only at tectonic plate boundaries during continental collision or during deep subduction.

Compositional similarities between TTG and volcanic adakites (Fig. 1a), a rare product of modern subduction settings, have fueled arguments that TTG formed during melting of deeply subducted oceanic crust^8–10,14,16^. However, it has been argued that the thermal state of early Earth did not allow for subduction as we know it today^17^, or for efficient lithosphere thickening^18^, and that HP TTG were extracted from gravitationally unstable lower crust as it dripped into the mantle^16,19,20^.

Here we show that whereas HP TTG probably did originate at high pressures, the chemical signatures used to infer this more likely reflect source enrichment rather than high melting pressures, and this source was metasomatized lithospheric mantle—not any form (subducted, dripped) of lower crust. This means that formation of MP TTG reflects the deepest levels of crustal melting in the Archean—a process that did not require depths significantly greater than the ~40 km average thickness of modern continental crust.

## Results

**Fig. 2:**
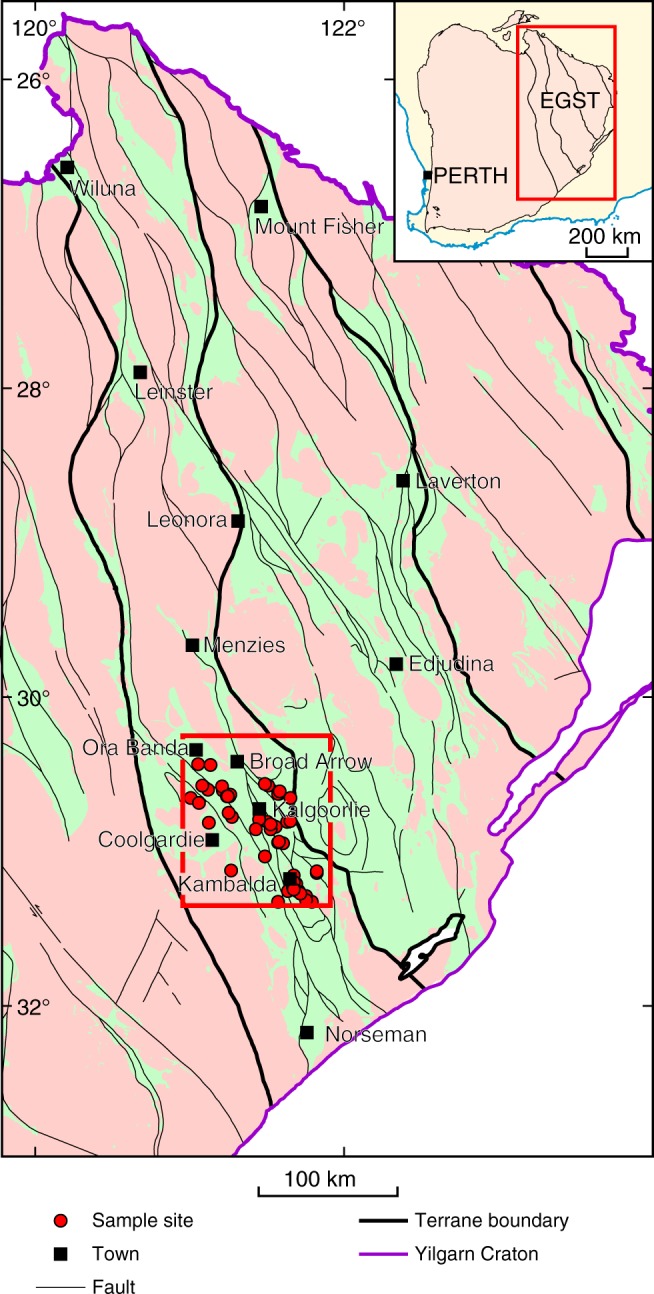
Location of samples within the Eastern Goldfields Superterrane (EGST). Inset outlining the Archean Yilgarn Craton within the southwest of Western Australia. The main map is a simplified geological map of the EGST showing greenstone belts in green and granitic rocks in pink and the locations of Black Flag Group samples used for this study. Note that many sites represent the location of diamond drill cores from which numerous samples were taken.

**Fig. 3:**
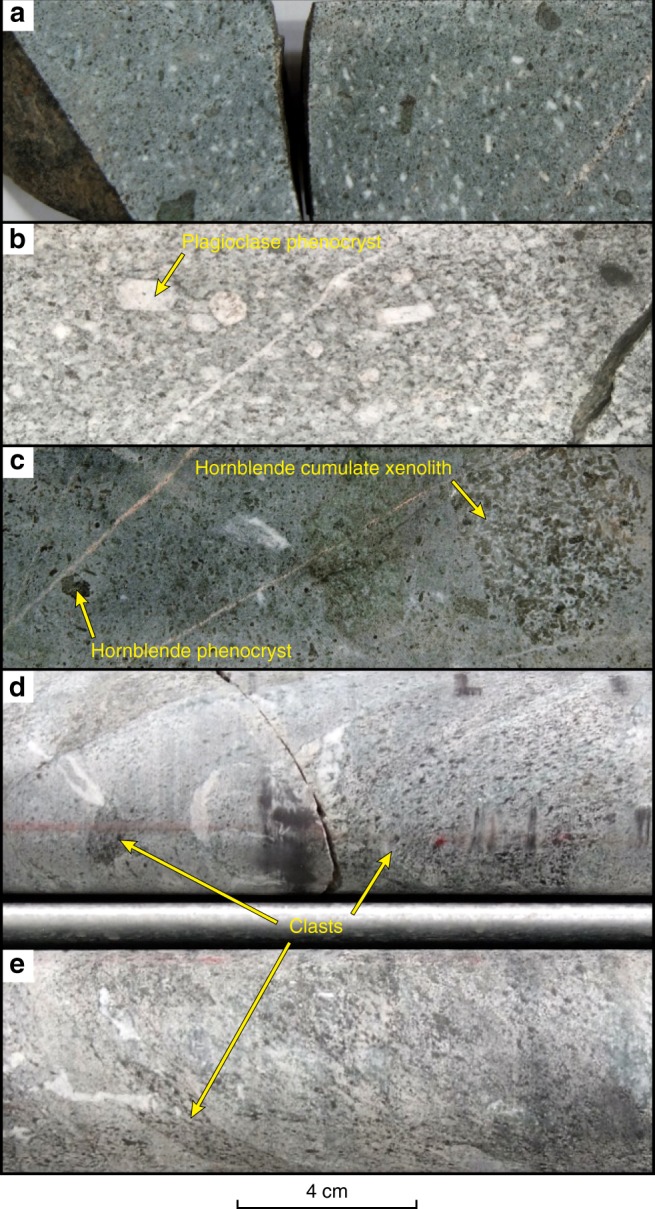
Photographs of sanukitoid in drill core from the Kalgoorlie to Kambalda region. **a** A fine-grained and relatively primitive volcaniclastic rock from the Black Flag Group (BFG); **b** an evolved and strongly porphyritic subvolcanic sanukitoid intrusion; **c** a primitive subvolcanic sanukitoid intrusion containing hornblende phenocrysts and hornblende cumulate xenoliths; **d, e** volcaniclastic breccia comprising clasts or fragments on fine- to medium-grained hornblende-porphyritic BFG in a matrix of fine-grained BFG.

Here, we study a geochemical dataset (Supplementary Data 1) of Neoarchean felsic volcanic and sub-volcanic rocks from the Kalgoorlie region of the c 2.7–2.64 Ga Eastern Goldfields Superterrane (EGST) of the Yilgarn Craton, Western Australia (Fig. 2). The EGST is a classic Archean granite–greenstone terrane, comprising a basalt-dominated supracrustal greenstone succession with komatiite, overlain by a dominantly felsic volcanic and volcaniclastic succession. These overlie and are intruded by granitic rocks broadly subdivided into TTGs that were mostly emplaced between 2.69 and 2.66 Ga, and more potassic monzodioritic to syenogranitic rocks largely formed through re-melting of pre-existing crust, including TTG, most of which were emplaced between 2.66 and 2.63 Ga^21,22^. Within the Kalgoorlie region, the felsic supracrustal succession is the 2.68–2.66 Ga^23^ Black Flag Group (BFG), which is dominated by sodic volcanic and volcaniclastic rocks and compositionally identical hornblende–plagioclase porphyritic subvolcanic intrusions. The stratigraphy underlying the BFG is locally intruded by hornblende ± plagioclase porphyritic dykes of sodic high-Mg diorite called sanukitoid (Fig. 3), with magmatic crystallization ages that overlap those of the BFG^24^. Rocks of the BFG all record a variable greenschist facies metamorphic overprint. Therefore, our geochemical data has been filtered to minimize the cryptic geochemical effects of metamorphism and hydrothermal alteration (see Methods).

### Chemical characteristics of sanukitoids

**Fig. 4:**
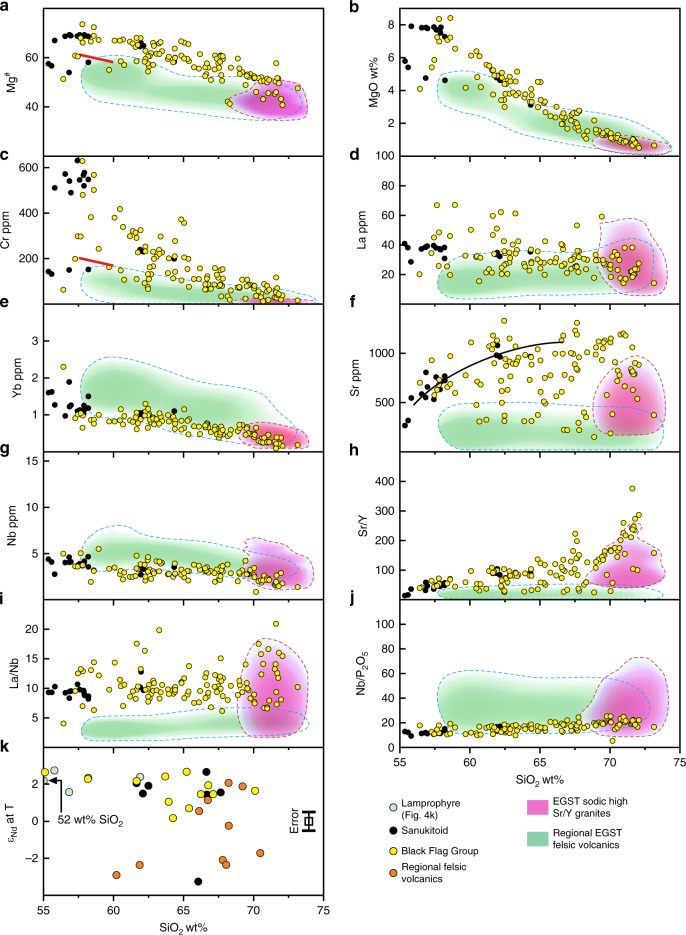
Compositional comparisons between the Black Flag Group (BFG) and contemporaneous regional felsic volcanics. Variations in selected major and trace elements, trace element ratios (Mg^#^ (**a**), MgO (**b**), Cr (**c**), La (**d**), Yb (**e**), Sr (**f**), Nb (**g**), Sr/Y (**h**), La/Nb (**i**) and Nb/P_2_O_5_ (**j**)) and initial Nd isotope compositions (**k**) with SiO_2_ (calculated volatile free) for rocks of the BFG (yellow dots), and regional felsic volcanic rocks from throughout the Eastern Goldfields Supertarrane (EGST) (green field), and sodic TTG of the EGST (purple field). Nd isotope data are plotted in *Ɛ* notation and calculated at 2.69 Ga (see Supplementary Table 2) (maximum error in Nd isotope determinations equate to ±0.5 *Ɛ* units—see error bar in Fig. 4k). Dashed lines outline kernel density plots comprising 90% of the data (see Methods). Red lines in **a** and **c** are the lower limit for sanukitoid at ~60 wt% SiO_2_ (ref. ^25^). Contemporaneous sanukitoid intrusions (black dots) into the stratigraphy beneath the BFG are also shown. These are typically less altered than their volcanic equivalents and show better constrained trends for Sr, although this follows the trend of highest data density within the filtered BFG dataset, suggesting the scatter in BFG data at lower Sr concentration reflects plagioclase-destructive alteration that filtering has not removed. The regional felsic volcanic data include the volcanic equivalents of the main intrusive (granitic) suites recognized throughout the EGST^29^ (see also Fig. 5). Unlike many sanukitoids, the BFG have low Nb concentrations with wide ranges in La/Nb, mainly reflecting variable La at relatively constant Nb. This points more to source enrichment through fluid rather than melt-metasomatism.

Sanukitoids have high concentrations of light rare-earth elements (LREE), Sr and Ba and high Sr/Y and La/Yb ratios like TTG, but additionally have high MgO, Cr and Ni contents, many with Mg^#^ >60^(25)^ (Fig. 1) reflecting compositions that equilibrated with mantle peridotite. Hence, rather than melting of basaltic crust, sanukitoid is thought to have formed through low-degree partial melting of the lithospheric mantle^25^. The sanukitoid underlying the BFG, for example, has the same radiogenic Nd isotopic composition as spatially and temporally related^25^, and geochemically similar, mantle-derived lamprophyre (Figs. 1 and 4). As with lamprophyre, the enriched geochemical features in primitive sanukitoid, or their parental magmas, are thought to reflect earlier enrichment of a lithospheric mantle source in incompatible trace elements, possibly by fluids or melts derived from delaminating lower crust or subducting oceanic crust^25–27^.

### Petrogenetic affinities of the BFG

**Fig. 5:**
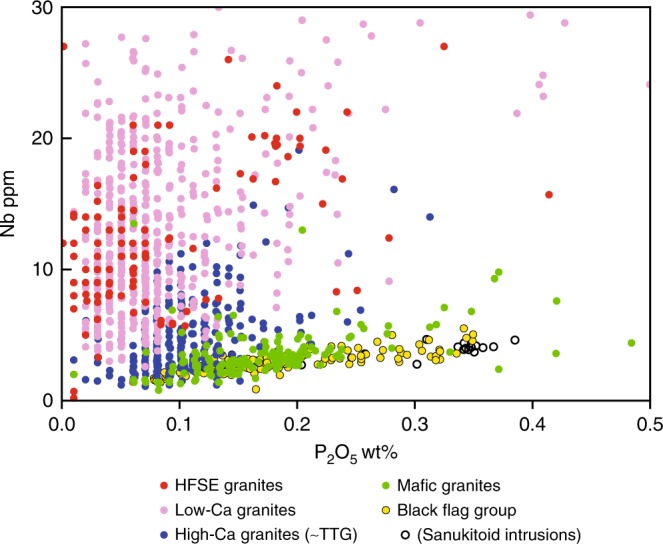
Compositional comparison between sanukitoids and other granitic rocks of the Eastern Goldfields Supertarrane. Variation in Nb concentration with concentrations of P_2_O_5_, comparing sanukitoids (including the volcanic rocks of the Black Flag Group) with granites of the eastern Yilgarn Craton. Granites in the Yilgarn Craton have been divided between two main groups^29^ (High-Ca and Low-Ca granites) and two minor groups (high field-strength element (HFSE) granites and mafic granites). See Data Availability for the source of geochemical data for the eastern Yilgarn Craton granites.

Because of broad compositional similarities with TTG, the BFG has been attributed to melting of deeply subducted basaltic crust^28^ (Fig. 1). However, our data indicate a different petrogenesis. The BFG volcanic rocks form a continuous compositional array extending over a significant silica interval (56–73 wt%), and most have much higher Mg^#^ and higher concentrations of MgO, Cr, Ni, LREE and Sr than other EGST felsic magmas at silica contents below ~68 wt% (Fig. 4). The BFG volcanic rocks are eruptive or near-eruptive sodic sanukitoids and, at low silica contents, are geochemically and isotopically identical to the age-equivalent sanukitoid intrusions from the underlying stratigraphy (Fig. 4). The volcanic rocks are the evolved equivalents of the sanukitoid intrusions and represent the first confirmed occurrence of sanukitoid volcanics. The BFG (including the intrusive sanukitoids) can also be distinguished by their high La/Nb and low Nb/P ratios (Fig. 4) from all other felsic rocks of the EGST. Two exceptions include a small number of high silica (>70 wt%) sodic, high Sr/Y, TTG-like rocks, which possibly also represent evolved sanukitoid, and rare hornblende-bearing granites (referred to as the mafic granites^29^), the most abundant high-Sr subset of which are also sanukitoids^29^ (Fig. 5).

**Fig. 6:**
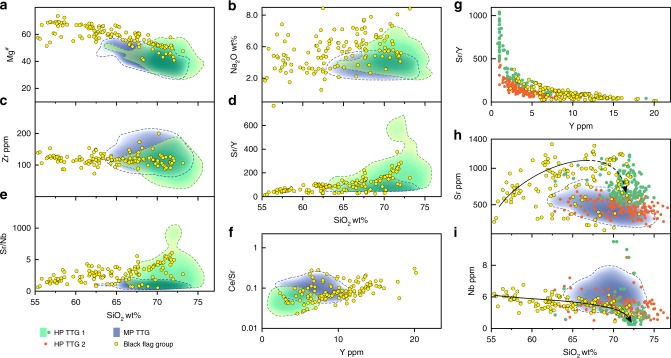
Compositional comparisons between the Black Flag Group (BFG) and global high pressure TTG and medium pressure TTG. Variations in selected major and trace elements and trace element ratios (Mg^#^ (**a**), Na_2_O (**b**), Zr (**c**), Sr/Y (**d**), Sr/Nb (**e**)] with SiO_2_ (calculated volatile free), Ce/Sr (**f**) and Sr/Y (**g**) with Y, and Sr (**h**) and Nb (**i**) with SiO_2_ (calculated volatile free). Dashed lines in **a**–**h** outline kernel density plots comprising 90% of the data (see Methods). Trends lines in **h** and **i** are approximations of highest data density. High pressure (HP) TTG forms the evolved end member of the BFG array, critically also in terms of the compositional attribute used to distinguish medium-pressure (MP) and HP TTG. HP TTG can clearly be subdivided compositionally (6g) – one subdivision (HP TTG 1) being characterized by very high-Sr and typically low-Nb, is intersected by the BFG trend. The second subdivision (HP TTG 2), is distinct from silica-rich BFG but indistinguishable from MP TTG in terms of (for example) Sr and Nb (used to infer stability/instability of plagioclase and rutile, respectively).

The expanded BFG compositional array is also distinct from global MP TTG, which has lower Mg^#^, is typically less sodic and is relatively enriched in Nb and possibly Zr (Fig. 6). Notably, at silica contents >68 wt%, the BFG array evolves directly into the field of HP TTG (Figs. 1, 4 and 6). However, although formed at mantle depths, the composition of silica-rich BFG is not a reflection of melting at high pressure. Primitive BFG, with MgO as high as 8.4 wt% and Mg^#^ >73, already contained ~600 ppm Sr and ~40 ppm La. Their source was a hydrous plagioclase-poor (or absent) peridotite. High Sr concentrations and Sr/Y ratios, therefore, cannot be related simply to melting of plagioclase, but were an intrinsic feature of this source, which was lithospheric mantle, not crust. The continuity of the BFG compositional array over such a wide silica interval strongly suggests that the composition of silica-rich BFG samples was achieved through fractional crystallization of primitive BFG magmas.

### Hornblende fractionation of metasomatized mantle melts

Previous studies^30^ have suggested that the lamprophyre and sanukitoid intrusions underlying the BFG are related through hornblende fractionation. Such a direct genetic relationship is consistent with our data, which show these rocks to be mineralogically transitional, spatially and temporally related^25^, and to have virtually identical geochemical and Nd isotope compositions (Fig. 4). Hydrous and incompatible trace element-enriched compositions show both lamprophyres and primitive (~8 wt% MgO; Mg^#^ >70) sanukitoid to reflect very low-degree partial melting of trace element-enriched peridotite, irrespective of whether they are directly related magma series.

Experimental work on mantle peridotite under water-saturated conditions^31,32^ has demonstrated the stability of amphibole up to 3 GPa and 1050 °C, and the likelihood of subduction-related metasomatizm forming hornblende-rich lherzolite at depth as shallow as ~50 km, with a zone of maximum water-storage capacity at depths of 80–100 km. Experimental data also show that hornblende joins the liquidus assemblage before olivine in hydrated (>6.5 wt% dissolved H_2_O) mantle-derived mafic magmas^33^. These results are consistent with requirements that sanukitoid crystallized from magmas with >7 wt% dissolved H_2_O (ref. ^34^), and with the common occurrence of amphibole cumulates in the sanukitoid of the Kalgoorlie area (Fig. 3) and elsewhere^34^.

**Fig. 7:**
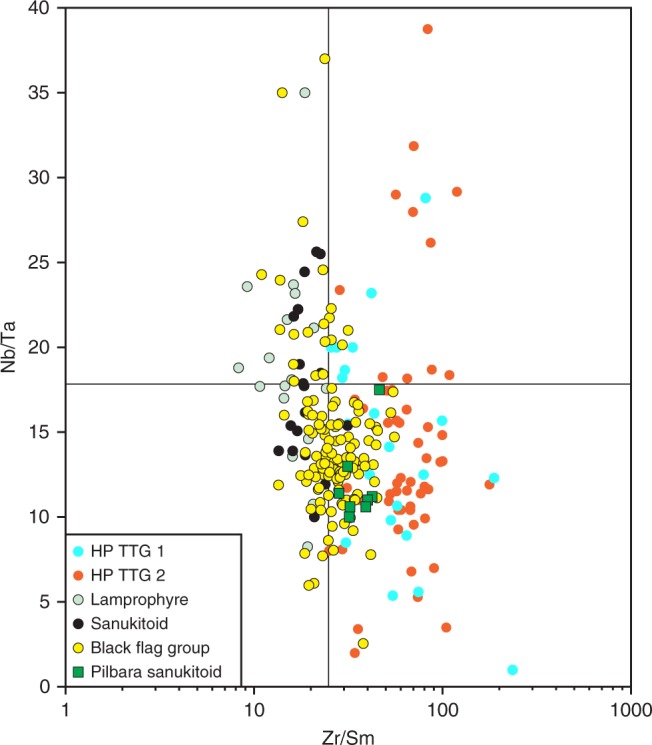
Plot of Nb/Ta against Zr/Sm comparing data from the Black Flag Group, and associated rocks, with high-pressure TTG. When plotted on a Nb/Ta vs. Zr/Sm^35^ diagram, TTG show a wide scatter at typically low Nb/Ta and high/Zr ratios. Lamprophyres, intrusive sanukitoids and volcanic rocks of the Black Flag Group show a trend with increasing compositional evolution, towards the field of high-pressure (HP) TTG. Sanukitoids from the Pilbara Craton^27^ are also plotted for comparison.

A plot of Nb/Ta against Zr/Sm (Fig. 7) has been used to emphasize the need for an amphibole-rich residual mineralogy during melting of a mafic source to form TTG^35^, which typically plot in the high Zr/Sm, low Nb/Ta field because hornblende, at Mg^#^ <70, strongly partitions Sm and Nb in preference to Zr and Ta. The Kambalda lamprophyres and sanukitoid intrusions studied here have lower Zr/Sm and higher Nb/Ta and, unsurprisingly, plot in the field expected for arc magmas. The Nb/Ta ratios for the primitive rocks scatter widely around primitive mantle values possibly because high Mg^#^ amphibole in ultramafic source rocks does not effectively fractionate Nb from Ta^35^ or because amphibole was exhausted during melting of the peridotitic source. Irrespective, the combined data for the lamprophyres, intrusive sanukitoids and BFG shows a distinct evolutionary trend to higher Zr/Sm and lower Nb/Ta that strongly implicates hornblende fractionation and, as seen previously (Figs. 1, 4 and 6), drives compositions into the field for HP TTG. A significant control on the low Zr/Sm ratios in many of the primitive BFG and associated rocks might be the LREE-enriched metasomatic additions to otherwise incompatible trace element poor peridotitic source compositions. Sanukitoids from the Pilbara Craton^27^, with similar silica contents to those from the EGST, have higher Zr/Sm (and La/Nb) ratios perhaps indicating a more significant silicate melt contribution to the metasomatic addition (see also Fig. 4).

**Fig. 8:**
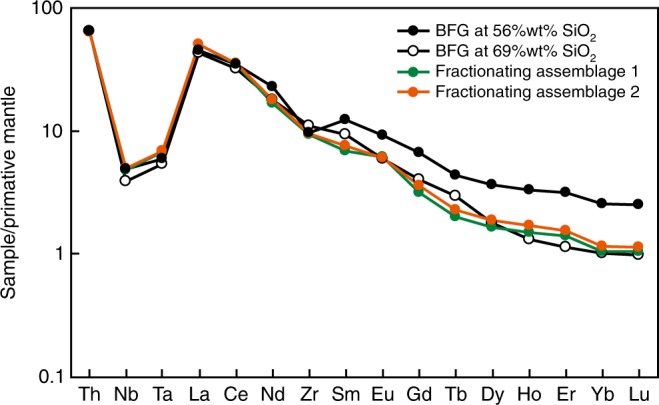
Results of trace element modeling. Primitive mantle normalized trace element diagram showing the modeled parental magma (56 wt% SiO_2_ – Supplementary Table 1) and comparing the compositions of evolved melts resulting from removal of two plausible fractionating mineral assemblages with evolved BFG liquids at 69 wt% SiO_2_. Mineral/melt distribution coefficients (*D*; Supplementary Table 1) are from refs. ^13,56^ except that for hornblende we use a *D*_Nb_ value of 0.8^39^.

We tested whether the BFG compositional array itself could also reflect fractional crystallization of a hornblende-rich assemblage (Fig. 8). In view of the scatter in data for some trace elements, we computed linear best-fits to the data and verified these models against trends in kernel density diagrams. In this way, we estimated the compositions of a primitive and evolved BFG sanukitoid in a single evolution interval from 56 to 69 wt% SiO_2_ (Supplementary Table 1).

Only models involving Rayleigh fractional crystallization of hornblende-dominated fractionation assemblages, with minor apatite and accessory zircon, closely matched the target compositions (Fig. 8), and the required 20 to 30% crystallization. These crystallization estimates are consistent with the modeled increase in silica from 56 to 69 wt% and demonstrate the strong affect that fractionation of silica-poor minerals such as hornblende (~45 wt% SiO_2_) have on expediting the transition from mafic to felsic magma compositions. Close matches between model and target compositions were obtained for 30% removal of an assemblage comprising 96.8% hornblende, 3% apatite and 0.2% zircon (Supplementary Table 1), although the modeled melts developed a slight positive Eu-anomaly. Addition of a small amount of plagioclase to the assemblage (81.7% hornblende, 15% plagioclase, 3% apatite and 0.3% zircon) reduced the effect that amphibole removal had on Eu and produced overall tighter fits with the model target liquids. Concentrations of LREE, Nb and Ta increased marginally instead of showing a predicted constant to marginal decrease in concentration but remained well within the natural array of the BFG. For the modeled fractionating assemblages, the bulk distribution coefficients for Nb (Ʃ*D*_Nb_) are close to unity (1.02 and 0.90). Several studies of calc-alkaline magmas indicate *D*_Nb_ values for amphibole higher^36–38^ than the value selected here (0.8 (ref. ^39^)), ranging to 2.5, which might account for the observed trends for Nb, including the inflection to decreasing values at SiO_2_ >68 wt%, although this might also reflect the late onset of zircon fractionation (zircon *D*_Nb_ ~ 50; Supplementary Table 1) at a similar SiO_2_ concentration (Supplementary Fig. 1). Hornblende and apatite preferentially incorporate Dy over Yb (i.e., *D*_Dy_ > *D*_Yb_; Supplementary Table 1) and so fractionation of these minerals should lead to decreasing Dy/Yb in residual magmas (considered the hallmark of hornblende fractionation^40^). Both the BFG compositional array and our model results, show a very slight overall increase in Dy/Yb, although this ratio initially decreases in the BFG array over a wide silica interval up to ~68 wt% SiO_2_ (Supplementary Fig. 1), where subsequent increase might again reflect the onset of zircon saturation and removal (*D*_Dy_ « *D*_Yb_ in zircon; Supplementary Table 1).

Modeling evolution at SiO_2_ contents >69 wt% was not attempted because of the added complications that significant removal of plagioclase and zircon (and potentially other accessory minerals) might have, as heralded by downward inflections in Sr and possibly Zr concentrations at SiO_2_ >66 wt%. Although the data for Sr are very scattered, the combined data for the sanukitoid intrusions and the broad trend in data for the BFG data does indicate a relatively strong initial increase in Sr concentrations to a plagioclase saturation point at ~66 wt% SiO_2_ and at ~1100 ppm Sr, followed by a rapid decrease in Sr concentration. For a primitive magma with ~56 wt% SiO_2_ and ~700 ppm Sr, and assuming Sr is perfectly incompatible (i.e., plagioclase is unstable), concentrations of ~1,100 ppm Sr are attained through only slightly more than 25% crystallization. Hence, the requirement for a minor amount of plagioclase fractionation is realistic, providing this occurs relatively late in the crystallization history (i.e., reflects only the evolution interval after the Sr inflection at ~66 wt% SiO_2_).

### HP TTG are hornblende-fractionated mantle melts

Hornblende-dominated fractionation of hydrous mantle-derived calc-alkaline basaltic melts has long been advanced as an alternative to deep crustal garnet-present melting for the formation of sodic felsic magmas with the high Sr/Y and La/Yb ratios of adakite or TTG^41^. These ideas have typically been rejected for TTG, primarily because neither the lower-silica segment of such a putative liquid line of descent (or crystallization path) nor the complimentary hornblende ± garnet cumulates are commonly recognized in the fragmented record of Archean terranes^6^. The absence of appropriate residual mineral assemblages is a problem regardless of the model chosen to explain TTG genesis but suggests that the lower-crustal segments where these rocks formed is nowhere exposed or preserved. In terms of the required liquid line of descent, the greenstones of the Kalgoorlie region are among the most studied major Archean supracrustal sequences on Earth. Here, the full high Sr/Y sodic liquid line of descent connecting primitive sanukitoid magmas with Mg^#^ >70 to HP-TTG-like compositions forms one of the most conspicuous stratigraphic components of the regional greenstone sequence but, to date, is a feature that has not been recognized. Removing amphibole, with only ~45 wt% SiO_2_ can drive a BFG-type magma from ~56 to ~70 wt% SiO_2_ through <30% crystallization, consistent with our trace element modeling. Consequently, intermediate links in a liquid line of descent, here and in other Archean terranes, might simply have been overlooked. If evidence for the primitive stages of a liquid line of descent is not available or not recognized, an assumption that evolved (i.e., SiO_2_ >70 wt%) rocks reflect melting of mafic crust at very high pressure could incorrectly be made.

A closer comparison with the global TTG data shows that the evolved end of the BFG array consistently intersects only a specific, albeit significant, part of the HP TTG field (Fig. 6) (high-Sr TTG). This is well demonstrated on a plot of Sr/Y vs. Y (Fig. 6g), but these sodic, (trondhjemitic), fractionated BFG rocks all carry critical compositional attributes of TTG; most samples having K_2_O/Na_2_O < 0.5, Sr/Y > 100, Sr > 700, Yb < 0.4 ppm, and La/Yb > 60 at SiO_2_ > 70 wt%, with a negligible or no Eu-anomaly. The remainder (second group) of the HP TTG samples typically lie within or marginal to the field for MP TTG for most compositional criteria, including elements such as Sr and Nb, used to infer the stability of pressure-sensitive minerals such as plagioclase and rutile. The trend for Sr vs. SiO_2_ (Fig. 6h) for the second group closely replicates that for MP TTG and is distinct from the steep trend shown by the high-Sr TTG. Members of the second group are probably better regarded as MP TTG than as very high-pressure crustal melts. Likewise, it is also very unclear that the high-Sr TTG reflect high-pressure crustal melts. Like the BFG rocks, these rocks clearly have compositions consistent with melting of metasomatized mantle lithosphere, and subsequent hornblende-dominated fractionation. The high-Sr TTG might reflect cases where an apparent absence of evidence for the primitive stages of a liquid line of descent has resulted in incorrect petrogenetic interpretations. What is also apparent is that the abundance of TTG that could be interpreted as very high-pressure crustal melts, based on geochemistry, has likely been significantly overestimated.

**Fig. 9:**
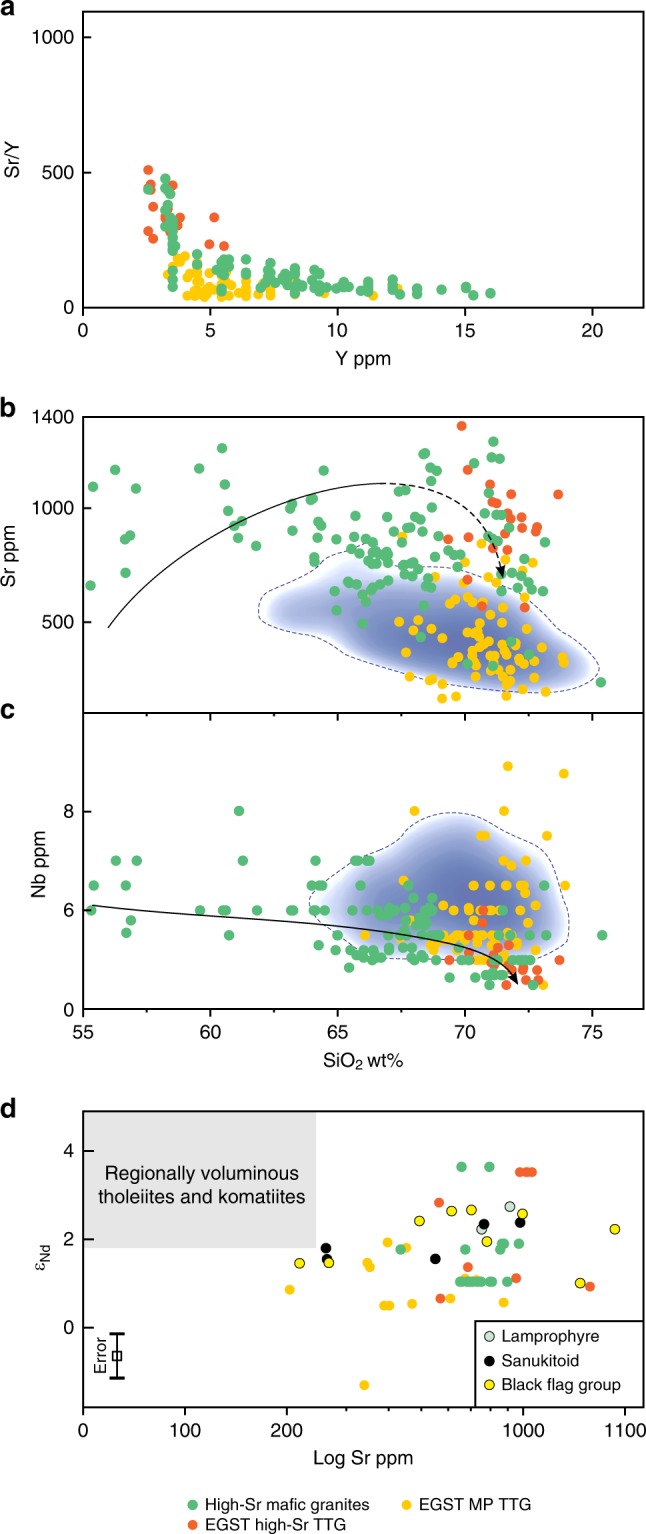
Compositional comparisons between the high-Sr mafic granites and various TTG-like intrusions in the Eastern Goldfields Superterrane (EGST). **a**, As with Fig. 6g, we use variations in Sr/Y vs. Y to identify a group of EGST TTG that lie on the same trend as sanukitoid (in this case the high-Sr subset of the regional mafic granites^42^ see Data Availability) – the EGST high-Sr TTG. **b**, **c**, EGST high-Sr TTG, like many rocks classified as high pressure (HP) TTG, lie at the evolved end of the liquid line of descent defined by the Black Flag Group (BFG) (black lines) and the high-Sr mafic granites, whereas the remainder of the EGST TTG fall into the field of medium pressure (MP) TTG (blue field as from Fig. 6) based on a range of compositional criteria (c.f. Figure 6h, i); **d**, Nd-isotope data plotted against Sr concentration showing the range of overlap, at primitive or radiogenic isotope ratios, between the high-Sr mafic granites and the EGST high-Sr TTG, and the limited overlap with EGST MP TTG, which trend to lower *Ɛ*_Nd_ at lower Sr concentrations. Also shown is the Nd-isotope range for the BFG and associated sanukitoid and lamprophyre intrusions as well as for regional basaltic and komatiitic rocks^43^. Nd-isotope data are from refs. ^43,60^. Maximum error in Nd isotope determinations equate to ±0.5 Ɛ units—see error bar in Fig. 9d).

We further examine potential genetic links between sanukitoid and rocks with HP TTG-like signatures by considering Nd isotopic compositional variations in c. 2.66–2.70 Ga granitic rocks from the Eastern Goldfields Superterrane that either fall within the high-Sr field of the hornblende-bearing mafic granites (regional sanukitoid plutons)^21,29,42^, or that can be confidently classified as TTG (i.e., K_2_O/Na_2_O < 0.6, Sr/Y and La/Yb both > 40; see Methods), none of which were available to the global TTG dataset of ref. ^9^. The EGST TTG were subdivided in the same way as the global HP TTG data set. This produced a high Sr/Y subgroup (EGST high-Sr TTG) with HP TTG-like signatures and that follows the trend defined by the high-Sr mafic granites, and a second, lower Sr/Y, subgroup that closely overlaps the field for MP TTG (EGST MP TTG subgroup; Fig. 9a). The former subgroup is compositionally indistinguishable from the high-Sr TTG subgroup of the global HP TTG data that followed the BFG hornblende fractionation trend (Fig. 9). It also shows a broad range of radiogenic Nd isotope compositions (Ɛ_Nd_ +1.0 to +3.6) that approaches Depleted Mantle values and that completely encompasses the range for the BFG (Ɛ_Nd_ mostly +1.5 to +2.7), sanukitoid and lamprophyre intrusions from the stratigraphy underlying the BFG (Ɛ_Nd_ mostly +1.6 to +2.7), as well as extensively overlapping the range for the most radiogenic basaltic and komatiitic units of the regional greenstone stratigraphy (Ɛ_Nd_ +1.8 to +5.4 (ref. ^43^)) (Fig. 9d). This isotopic range is largely distinct from that of the generally less radiogenic EGST MP TTG (Ɛ_Nd_ +1.5 to –1.3), which clearly requires a petrogenesis involving greater interaction with isotopically evolved crust.

In the case of the EGST high-Sr TTG, requirements that the mafic source for TTG was significantly more enriched in incompatible trace elements than typical Archean tholeiite^7,44^, rules out melting of lower crustal equivalents to the MORB-like tholeiitic magmas that dominate all eastern Yilgarn greenstone sequences, unless that bulk source also incorporated a Th- and LREE-enriched crustal component, which our Nd isotope data do not permit. The EGST high-Sr TTG are compositionally and isotopically equivalent to the evolved BFG and the mafic granites which evolved through hornblende-dominated fractionation. Based on the high MgO content and Mg^#^ (up to 8.4 wt% and 73, respectively) of primitive BFG rocks and on their isotopic and compositional equivalence with temporally and spatially associated lamprophyres (MgO up to 11.7 wt%, Mg^#^ up to 73), primitive BFG magmas were partial melts of incompatible trace element enriched and hydrated peridotite. Average depleted mantle model ages (T_2DM_^45^—the average mantle extraction age of all components in the bulk source), ~2.83 Ga for the lamprophyres and ~2.86 Ga for the BFG, are close to the magmatic ages and indicate metasomatic source enrichment occurred only shortly before magmatism.

## Discussion

Rocks that have been classified as HP TTG form <25% of global TTG data^9^ but are considerably less common in Australian Archean cratons (<3% in the Pilbara Craton; no data for the Yilgarn Craton). We suggest the global figure is a significant overestimate. It comprises rocks that are better classified as MP TTG that did not form through melting at extraordinary depths, and fractionated sodic sanukitoids representing relatively small volume magmas derived through melting of enriched mantle lithosphere, not of mafic crust. Thus, there is no clear evidence for melting of Archean mafic crust at extraordinary depths.

**Fig. 10:**
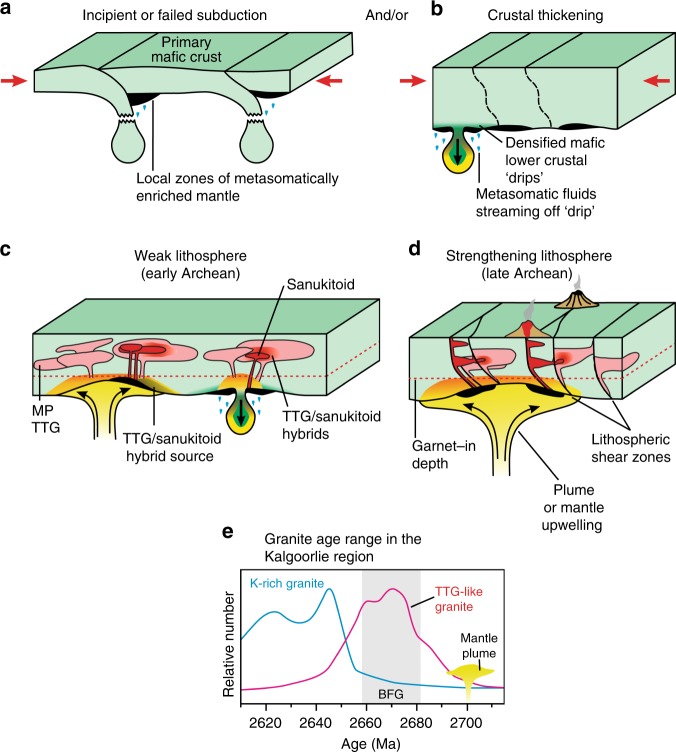
Schematic model showing the geological environment of TTG and sanukitoid formation. Block diagrams illustrating potential sites of Archean medium pressure (MP) TTG and sanukitoid (including high-pressure (HP) TTG) formation. Pockets of metasomatized lithospheric mantle (MLM) representing the source for sanukitoid form throughout the Archean during incipient, or failed, mafic oceanic-plate subduction (**a**) and possibly also after thickening (due to greenstone accumulation and/or crustal shortening) when densified and gravitationally unstable lower mafic crust dripped into the mantle, releasing volatile components (**b**). Melting of primary mafic crust below the garnet-in zone (red line), was induced by mantle upwelling. A ductile crust in the early Archean (**c**) meant that sanukitoid melts (red) derived from remnant MLM sources could not effectively ascend and evolve uncontaminated by crust and contemporaneous MP TTG (pink) crustal melts. The development of lithosphere-scale structures in Neoarchean crust (**d**) and possibly an increase in the volume/number of MLM sources during the transition to a global plate tectonic regime, increased both the proportion of sanukitoid magmatism and the efficiency of it rising to higher crustal levels without contamination. Where thermal anomalies were local or weak, only MLM was remobilised and sanukitoid magmatism was unassociated with MP TTG^27^. The Black Flag Group formed in the wake of widespread plume activity^23^ and MLM melting was synchronous with widespread lower crustal melting and MP TTG magmatism (TTG-like rocks (**e**) (data from ref. ^23^)).

Evidence for subduction in the Archean points more to short-lived periods of incipient, or failed, subduction^46–48^, ephemeral within a prevailing stagnant-lid regime^46^. This process, nevertheless, permits early slab-breakoff and the return of mafic material to the mantle^49^, required to balance the production of new primary mafic crust in other environments. Arguably more important in terms of recycling crust is dripping of gravitationally unstable residual assemblages of mafic lower crust that has already yielded a felsic melt^9,14,16^. Neither model necessarily relies on extraction of TTG melts during crustal recycling itself, but both environments have been suggested as sites where the inferred very high-pressure Archean felsic crust formed. Our evidence is that TTG has not been directly extracted from subducting or dripping mafic crust, and that HP TTG-like signatures do not form evidence for these recycling models. MP TTG reflect the deepest range of crustal melting during the Archean. These form through direct partial melting of mafic crust, which is a far more efficient means of producing the large felsic magma volumes typical of MP TTG than fractional crystallization^6,50^. Available data indicate that this melting occurred at depths of 25–45 km, still within the range of modern continental crust (35–40 km^14,15^). They nevertheless preserve evidence for garnet-present melting that is only rarely seen in granites from convergent margins, where modern felsic crust is formed^41^. Hence, we suggest that it is the composition and crustal level of melt zones that has changed through time, not crustal thickness (Fig. 10).

Post-Archean felsic crust forms in the mid- to lower crustal regions of continental arcs above subduction zones. Here, hydrated mantle-derived magmas intrude, crystallize and dehydrate, and melt and mix with pre-existing, evolved and compositionally heterogeneous crustal sources^51^. Melting conditions vary between those of water-fluxed melting^51^ and fluid-absent reactions consuming biotite, but hornblende-bearing felsic melts are common.

In contrast, the voluminous MP TTG that dominate Archean crust, and particularly the Archean cratons of Western Australia, typically contains biotite but little or no hornblende across a wide silica range^52,53^. Previous suggestions that TTG represented an Archean analog of modern hornblende-rich adakite^8–10,14,16^ highlighted a need for a significantly hydrated source. However, the dominance of biotite over hornblende reflects generally drier or more evolved source compositions^54,55^. Since the source of TTG was mafic, it seems likely that it was indeed typically not as hydrated as the source of the hornblende-rich felsic magmas commonly found in post-Archean subduction settings. Sanukitoid represents the main expression of Archean hydrous mafic to felsic melts, and are rare.

We suggest that in the absence of water-fluxing, crustal melting to form MP TTG occurred at or near the base of garnet-amphibolite crust at higher temperatures through reactions consuming hornblende in more homogeneous, plateau-like mafic crust^7,13^. This was likely driven by thermal perturbations, plumes or mantle upwellings, resulting from delamination or dripping of dense residuals produced during earlier melting events^19,20,56^.

In the case of the EGST, our data show the bulk source for MP TTG to be too evolved in terms of Nd isotopes and too enriched in incompatible trace elements to be tholeiitic material similar to the basaltic rocks that dominate the greenstone sequences. These data require a source that was contaminated with pre-existing felsic crust, potentially older TTG itself, and highlight a process whereby TTG simply represents one end member in a continuum^11,53^ of sodic to more potassic granites, incorporating Transitional TTG^52,53^, reflecting a range of melting conditions and a source comprising homogeneous mafic crust with varying proportions of pre-existing felsic material^11,52,53^. Significant volumes of isotopically primitive crust with high incompatible trace element concentrations, appropriate for the source of end member Archean TTG, have not been identified^7^. However, an intriguing possibility is that this source simply reflects tholeiitic lower crust contaminated, homogenized and weakly hydrated by primitive sanukitoid, in lower crustal magma chambers.

The source regions of sanukitoids themselves were probably a local result of mantle metasomatism resulting from localized incipient, or failed, subduction, or through dehydrating delaminating, or dripping, crust. The first recognized occurrence of upper-crustal sanukitoid at c. 2.95 Ga^27^ reflects a point where Neoarchean lithospheric strengthening^18^ allowed the development of lithospheric structures that facilitated efficient extraction, channeling and upper-crustal emplacement of such deep-sourced magma (Fig. 10b). Earlier, or in the absence of such structures, sanukitoid magmas mostly stagnated (Fig. 10a) and contributed to overlying lower-crustal melt zones and to the wide compositional diversity of MP TTG and Transitional TTG. This process may perhaps explain the cryptic incompatible element and volatile enriched components required in TTG source regions. Those sanukitoid magmas that did ascend to mid-crustal levels rapidly became felsic through amphibole-dominated fractionation and have potentially been misidentified as HP TTG.

## Methods

### Sample location

Samples of the BFG were collected from the Kalgoorlie-Kambalda area within a 70 × 200 km NW-trending belt bounded between longitudes 120.91° and 121.82° and latitudes –31.35° and –30.24°. Samples of regional felsic volcanic rocks are from sites throughout the EGST and specific locations are cited in Supplementary Data 2.

### Sample selection and data handling

Metamorphic recrystallization of greenstone lithologies at a minimum of greenschist facies is near ubiquitous in the EGST. Variable degrees of whole-rock silicification, and weak to moderate carbonate-sericite-epidote alteration of feldspars is also widespread. Nevertheless, all BFG samples collected specifically for this study are from diamond drill core and represent, based on visual inspection, the freshest material available. Many of these drill holes were sited on mineral exploration targets and so measures were taken to minimize the potential effects of hydrothermal alteration. In addition to excluding visually altered samples and veined samples, the following geochemical screens were employed. All samples with analytical loss on ignition (LOI) >5 wt% were removed. All samples with an aluminium saturation index >1.1 were removed. Samples with SiO_2_ (calculated on an anhydrous basis) >74 wt% were removed. At SiO_2_ (anhydrous) >60 wt%, only samples with K_2_O + Na_2_O > 4 wt% (anhydrous) were retained. Plotting the remaining data in terms of CaO vs SiO_2_ produced well constrained negative trend, but with distinct outliers constituting >5% of the dataset; these outliers were removed. These filters left 143 freshest samples (Supplementary Data 1) from an initial dataset of 205 analyses. Tightly constrained mantle-normalized trace element plots (Fig. 1) suggest that these measures have certainly minimized the effects of alteration within samples of the BFG. For typically alteration-mobile trace elements such as Sr, whose concentration variations are important for petrogenetic interpretation of TTG, adakites and sanukitoids and other rocks in which plagioclase is an important component, filtering still leaves a moderate scatter of data, but with a clear higher density cloud at high Sr- concentrations (Figs. 4f and 6c) which concentrates around the better-defined Sr vs. SiO_2_ trend for the less altered intrusive sanukitoids that are the intrusive equivalent of the BFG.

For realistic comparison with our new data, TTG data from ref. ^9^ was filtered to remove data with K_2_O/Na_2_O >0.6 and Sr/Y <40. The two major granite groups identified in the EGST have been termed high Ca granites and low Ca granites^21,29^. The former typically have K_2_O/Na_2_O <1.0 and are broadly equated with TTG. The latter show extreme enrichments in incompatible trace elements, generally have K_2_O/Na_2_O >1.0 and are melts of compositionally variable crust and were excluded from this work. Only those high Ca granites with K_2_O/Na_2_O <0.6 and Sr/Y >40 were used for comparison with our data. For global Precambrian sanukitoids, we use the dataset of ref. ^58^. Sanukitoids show a wide range in K_2_O/Na_2_O ratio reflecting variations in source enrichment, fractional crystallization and crustal contamination. Again, we restrict our discussion to sodic (K_2_O/Na_2_O <0.6) sanukitoid.

Kernel Density plots included in Figs. 1, 4, 6 and 9 were created using ioGAS version 7 software (REFLEX). The intensity of the shade increases with increasing data density (i.e., the number of data points within a given volume) and the outlined fields enclose 90% of all data points.

### Whole-rock geochemical analysis

New data included in this study were collected at Bureau Veritas, Perth, Western Australia. Samples were crushed in a plate jaw crusher and milled in a low-Cr steel mill to produce a pulp with a nominal particle size of 90% <75 µm. Major and minor elements (Si, Ti, Al, Cr, Fe, Mn, Mg, Ca, Sr, Ba, Na, K and P) were determined by X-ray fluorescence (XRF) spectrometry on a fused glass disk and loss on ignition was determined by thermogravimetric analysis. Trace elements (including Ag, As, Ba, Be, Bi, Cd, Ce, Co, Cr, Cs, Cu, Dy, Er, Eu, Ga, Gd, Ge, Hf, Ho, La, Lu, Nb, Nd, Ni, Pb, Pr, Rb, Sc, Sm, Sn, Sr, Ta, Tb, Th, Tl, Tm, U, V, W, Y, Yb, Zn and Zr) were measured by laser ablation ICP–MS on a fragment of each glass disk earlier used for XRF analysis. Data quality was monitored by blind insertion of sample duplicates, internal reference materials, and the certified reference material OREAS 24b. BV Minerals also included duplicate samples, certified reference materials (including OREAS 24b), and blanks. Total uncertainties for major elements are ≤1.5%, those for minor elements are <2.5% (at concentrations >0.1 wt.%) and those for most trace elements are ≤10% (Lu ±20%).

### Sm-Nd isotope analysis

Sm-Nd isotopic values were determined on whole-rock samples at the Géosciences Rennes Laboratory using a 7 collector Finnigan MAT-262 mass spectrometer. Samples were spiked with a ^149^Sm–^150^Nd solution and dissolved in a HF-HNO_3_ mixture. They were then dried and taken up with concentrated HCl. In each analytical session, the unknowns were analyzed together with the Ames Nd-1 Nd standard, which during the course of this study yielded an average ^143^Nd/^144^Nd value of 0.511948 (±5). All the analyses of the unknowns have been adjusted to the long-term value ^143^Nd/^144^Nd value of 0.511963 for Ames Nd-1. Mass fractionation was monitored and corrected using the value ^146^Nd/^144^Nd = 0.7219. Procedural blank analyses yielded values of 200 pg for Nd and are therefore considered to be negligible.

## Supplementary information

Supplementary Information

Description of Additional Supplementary Files

Supplementary Data 1

Supplementary Data 2

## Data Availability

All new geochemical and locational data for samples of the Black Flag Group are provide in Supplementary Data 1 and Nd-isotope data are provided in Supplementary Table 2. These data can also be downloaded form from the Geological Survey of Western Australia’s WACHEM database (http://geochem.dmp.wa.gov.au/geochem/) using the GeoChem Extract tool and selecting the Whole State option. The source for geochemical data for samples of the regional felsic volcanic rocks is given in Supplementary Data 2 and includes published data^59^ as well as data that can be downloaded using the given sample numbers from the Geological Survey of Western Australia’s WACHEM database (http://geochem.dmp.wa.gov.au/geochem/) using the GeoChem Extract tool and selecting the Whole State option, and from Geoscience Australia’s OZCHEM National Whole Rock Geochemistry Dataset [https://ecat.ga.gov.au/geonetwork/srv/eng/catalog.search#/metadata/65464]. Data for Yilgarn Craton granites, including mafic granites are also from Geoscience Australia’s OZCHEM National Whole Rock Geochemistry Dataset [https://ecat.ga.gov.au/geonetwork/srv/eng/catalog.search#/metadata/65464]. The high-Sr subset of the mafic granites includes the Liberty, Lanarkshire, Lawlers, New Celebration, Dinky Boys, Bonnievale, Victory (1 & 2) and Kambalda (1 & 2) intrusions, and geochemical data for these is available from refs. ^21,40^. Data for HP TTG and MP TTG are from ref. ^9^ but have been filtered to remove data with K_2_O/Na_2_O >0.6 and Sr/Y <40. Data for sodic sanukitoids are those samples from ref. ^58^ with K_2_O/Na_2_O <0.6.
