# Supplementary material for: No evidence for high-pressure melting of Earth’s crust in the Archean
Source: Nat Commun. 2019 Dec 5;10:5559. doi: 10.1038/s41467-019-13547-x (PMC6895241; doi:10.1038/s41467-019-13547-x)
Supplement: Supplementary file 1 — Supplementary Information [file 41467_2019_13547_MOESM1_ESM.pdf]

No evidence for high-pressure melting of Earth’s crust in the Archean

Smithies et al

Supplementary Figure 1

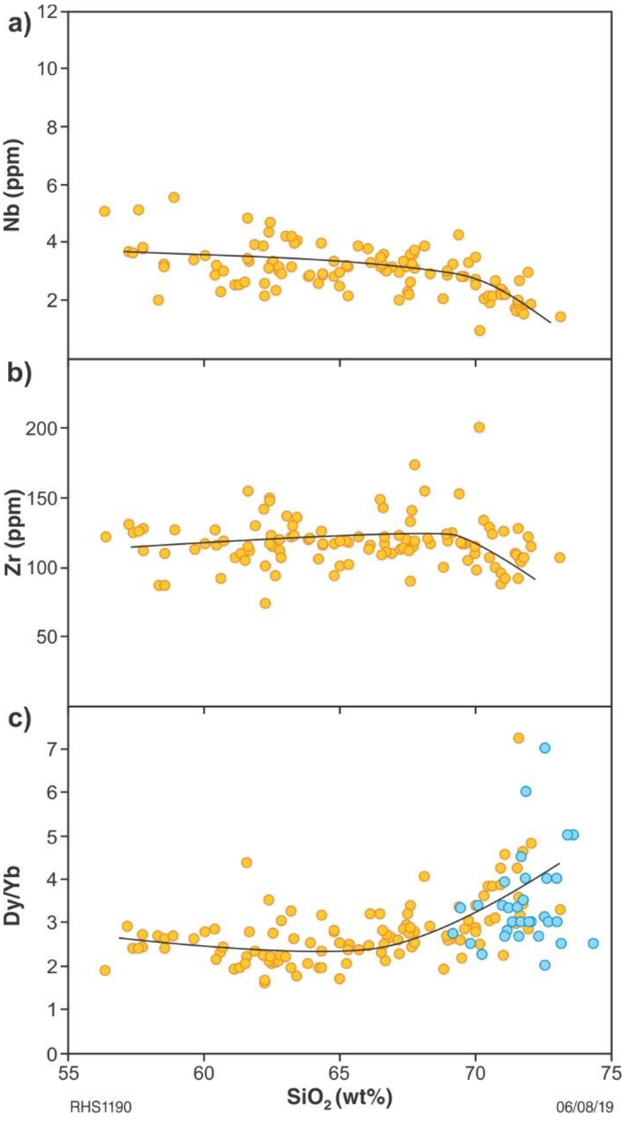

Supplementary Figure 1. Compositional variation diagrams showing variations in Nb concentration (a), Zr concentration (b) and Dy/Yb ratio (c) against silica for rocks of the Black Flag Group, and showing inflections potentially indicating late removal of zircon from fractionating magmas. Panel c) also includes data for the 'high-Sr TTG' subgroup of the global HP TTG data (blue dots)(see text Fig. 6g-i), suggesting that these magmas also undergo an increase, rather than decrease, in Dy/Yb ratio at high silica contents.

Supplementary Table 1. Rayleigh fractional crystallisation modelling.

Distribution coefficient (D)					Trace element concentration (p.p.m.)				
						Parental melt	Target composition	Model 1	Model2
	Amphibole	Plagioclase	Apatite	Zircon		BFG at 56 wt% SiO_2	BFG at 69 wt% SiO_2	Removal of Assemblage 1 (F=0.3)	Removal of Assemblage 2 (F=0.3)
Th	0.055	0.095	23	62	Th	5.47	5.42	5.39	5.36
Zr	0.417	0.078	0.9	130	Nb	3.45	2.76	3.42	3.53
Ta	0.38	0.053	0.05	50	Ta	0.24	0.22	0.28	0.28
Nb	0.8	0.239	0.05	50	La	30.54	29.15	34.39	34.31
La	0.319	0.358	12	26.6	Ce	61.45	56.26	61.63	62.36
Ce	0.56	0.339	15	23.5	Nd	30.58	24.12	22.63	23.91
Nd	1.32	0.289	19	21.7	Zr	107.62	122.18	104.62	106.53
Sm	2.09	0.237	20	17.7	Sm	5.43	4.14	3.05	3.37
Eu	1.79	2.17	13	12.1	Eu	1.54	0.99	1.03	1.01
Gd	2.53	0.192	20	15	Gd	3.94	2.37	1.88	2.13
Tb	2.6	0.17	19	37.3	Tb	0.47	0.32	0.21	0.24
Dy	2.55	0.15	18	60	Dy	2.67	1.3	1.21	1.37
Ho	2.41	0.132	16.8	120	Ho	0.54	0.21	0.25	0.28
Er	2.22	0.117	15.5	200	Er	1.5	0.54	0.66	0.74
Yb	1.79	0.094	13	490	Yb	1.25	0.5	0.51	0.56
Lu	1.59	0.085	10	632	Lu	0.18	0.07	0.08	0.08

Assemblage 1: 96.8% hornblende, 3% apatite and 0.2% zircon (F=0.3)

Assemblage 2: 81.7% hornblende, 15% plagioclase, 3% apatite, 0.3% zircon (F=0.3)

Mineral/melt distribution coefficients (D) are those used by refs. 13 and 56 except for hornblende for w hich w e use a DNb value of 0.8 (ref 39)

Supplementary Table 2. New Sm/Nb isotopic results

SeriesName	Sample ID	Latitude	Longitude	Sm (ppm)	Nd (ppm)	$^{147}Sm/^{144}N$	$^{143}Nd/^{144}Nd$	error (2se)	$(^{143}Nd/^{144}N$	εNd (t=2.69)	$T_{DM\ (Ga)}$	$T^2_{DM\ (Ga)}$
Lamprophyre	229908	-31.2978	121.7557	8.6	45	0.1156	0.511316	0.000005	0.509264	2.22	2.86	2.85
Lamprophyre	223135	-31.3263	121.7902	7.07	40.9	0.1045	0.511146	0.000004	0.509291	2.74	2.8	2.81
Sanukitoid	229912	-31.2978	121.7557	5.9	33.8	0.1055	0.511104	0.000005	0.509231	1.57	2.89	2.9
Sanukitoid	223138	-31.3263	121.7902	4.64	29.18	0.0961	0.510979	0.000005	0.509273	2.39	2.82	2.84
Sanukitoid	223249	-31.1775	121.6784	6	35.66	0.1017	0.511075	0.000005	0.509271	2.34	2.83	2.84
Sanukitoid	227954	-31.2112	121.6735	10.6	63.7	0.1004	0.510765	0.000005	0.508984	-3.28	3.2	3.26
Sanukitoid	211205	-31.2257	121.6746	4.83	29.74	0.0982	0.510994	0.000004	0.509252	1.98	2.85	2.87
Sanukitoid	211206	-31.2257	121.6746	3.7	19.78	0.1132	0.511239	0.000004	0.509231	1.56	2.9	2.9
Black Flag Group	204681	-30.619	121.2643	3	18.5	0.0994	0.510969	0.000005	0.509205	1.05	2.91	2.94
Black Flag Group	223074	-31.3263	121.7902	3.4	21.7	0.0959	0.510953	0.000005	0.50925	1.94	2.85	2.87
Black Flag Group	227909	-30.8236	121.5578	6.5	42.9	0.0912	0.510881	0.000005	0.509262	2.17	2.83	2.85
Black Flag Group	211547	-30.8155	121.5362	4.7	30.8	0.0921	0.510908	0.000005	0.509274	2.41	2.81	2.83
Black Flag Group	211529	-30.8155	121.5362	6.4	37.8	0.1022	0.511101	0.000005	0.509287	2.67	2.8	2.81
Black Flag Group	211535	-30.8155	121.5362	7.13	46.28	0.0931	0.510937	0.000005	0.509286	2.64	2.8	2.82
Black Flag Group	223068	-31.3263	121.7902	1.8	9.4	0.1153	0.511273	0.000005	0.509226	1.46	2.91	2.91
Black Flag Group	223086	-31.3263	121.7902	1.9	9.8	0.1159	0.511283	0.000005	0.509226	1.46	2.91	2.91
Black Flag Group	229913	-31.2978	121.7557	5.4	33.9	0.0956	0.510923	0.000005	0.509226	1.47	2.88	2.91
Black Flag Group	229904	-31.2978	121.7557	7.6	49.1	0.0939	0.510897	0.000005	0.509231	1.56	2.87	2.9
Black Flag Group	223141	-31.3263	121.7902	8.22	53.15	0.0935	0.510918	0.000005	0.509259	2.12	2.83	2.86
Black Flag Group	223153	-31.3263	121.7902	5.07	32.58	0.0941	0.510957	0.000005	0.509288	2.68	2.8	2.81
Regional Felsic volcanic	228126	-27.6025	120.5762	4.2	22	0.1161	0.511064	0.000005	0.509004	-2.9	3.26	3.23
Regional Felsic volcanic	228106	-27.602	120.5783	5.9	35.3	0.1013	0.510829	0.000005	0.509031	-2.36	3.15	3.19
Regional Felsic volcanic	230005	-26.862	121.5847	2.3	13.3	0.1039	0.510876	0.000005	0.509032	-2.35	3.16	3.19
Regional Felsic volcanic	229973	-26.7915	121.556	3.4	18.1	0.1148	0.511082	0.000005	0.509045	-2.09	3.19	3.17
Regional Felsic volcanic	228115	-27.6025	120.5762	3.5	21	0.1005	0.510848	0.000005	0.509064	-1.72	3.1	3.14
Regional Felsic volcanic	228078	-27.602	120.5783	2.8	18.4	0.0934	0.510797	0.000005	0.509139	-0.24	2.98	3.03
Regional Felsic volcanic	227918	-30.7953	121.6357	2.9	18.3	0.0941	0.51085	0.000004	0.50918	0.55	2.93	2.97
Regional Felsic volcanic	227917	-30.7953	121.6357	2.3	13.4	0.1047	0.511068	0.000005	0.509209	1.14	2.91	2.93
Regional Felsic volcanic	177923	-31.6219	122.0073	1.9	11.4	0.1029	0.511074	0.000005	0.509247	1.88	2.86	2.87
Regional Felsic volcanic	114144	-30.9767	122.0606	2.3	12.6	0.1099	0.511208	0.000005	0.509257	2.07	2.86	2.86
