# Supplementary material for: No evidence for high-pressure melting of Earth’s crust in the Archean
Source: Nat Commun. 2019 Dec 5;10:5559. doi: 10.1038/s41467-019-13547-x (PMC6895241; doi:10.1038/s41467-019-13547-x)
Supplement: Supplementary file 2 — Description of Additional Supplementary Files [file 41467_2019_13547_MOESM2_ESM.pdf]

Description of Additional Supplementary Files

File Name: Supplementary Data 1

Description: Whole rock major (wt%) and trace element (ppm) data.

File Name: Supplementary Data 2

Description: Data source for ‘regional felsic volcanic rocks’.
